# William Wilfrid King

**Published:** 1934

**Authors:** 


### WILLIAM WILFRID KING, F.R.C.S.

THE death of William Wilfrid King of Sheffield on July 9th in his fifty-second year is a heavy loss to British gynæcology. Born at Bath, he was educated at Stonyhurst and at Bristol Medical School, and qualified in 1906. He held the post of Resident Obstetric Officer at the Bristol Royal Infirmary, and for three years was House Surgeon at the Jessop Hospital for Women. He was appointed Clinical Pathologist to the Sheffield Royal Infirmary in 1911. There he worked particularly at the toxæmias of pregnancy, and in 1913 produced his first important paper, on the Abderhalden reaction. In 1920 he was elected Surgeon to the Jessop Hospital. He was a very active member of the North of England Obstetrical and Gynæcological Society, Secretary in 1923 and President in 1931.

Among other notable contributions to medicine are his work on pelvic adenomyomata and on droplet infection in relation to puerperal sepsis. Widely read, keenly observant and alert, he was always prepared to discuss any question relating to his specialty. His clinical teaching was admirable, and the loss to Sheffield University will be very deeply felt. Although very active and always at work, King was not a robust man; his professional work absorbed his energies, and he had no time or perhaps no patience for games or other forms of recreation. He leaves a widow and four children, to whom our sincerest sympathies are extended.

